# Prenatal Diagnosis of Bovine Aortic Arch Anatomic Variant

**DOI:** 10.3390/diagnostics12030624

**Published:** 2022-03-02

**Authors:** Liana Pleș, Cătălin Cîrstoveanu, Romina-Marina Sima, Gabriel-Petre Gorecki, Radu Chicea, Bashar Haj Hamoud

**Affiliations:** 1 Department of Obstetrics and Gynecology, ‘Carol Davila’ University of Medicine and Pharmacy, 020021 Bucharest, Romania; liana.ples@umfcd.ro; 2 The “Bucur” Maternity, ‘Saint John’ Hospital, 040294 Bucharest, Romania; 3 Department of Pediatrics, ‘Carol Davila’ University of Medicine and Pharmacy, 020021 Bucharest, Romania; catalin.cirstoveanu@umfcd.ro; 4 Pediatrics Department, ‘Maria Sklodowska Curie’ Emergency Children Clinical Hospital, 041451 Bucharest, Romania; 5 Faculty of Medicine, ‘Titu Maiorescu’ University, 040441 Bucharest, Romania; 6 Faculty of Medicine, ‘Lucian Blaga’ University of Sibiu, 550024 Sibiu, Romania; radu.chicea@gmail.com; 7 Department for Gynecology, Obstetrics and Reproductive Medicine, Saarland University Hospital, Kirrberger Straße 100, Building 9, 66421 Homburg, Germany; bashar.hajhamoud@uks.eu

**Keywords:** aortic arch variant, bovine aortic arch, prenatal diagnosis, cardiac surgery

## Abstract

Fetal aortic arch development is an early and complex process that depends on many genetic and environmental factors. The final aortic arch varies greatly; it may take the form of a normal arch, anatomic variant (AAAV) with a common origin to that of the innominate artery and left common carotid artery (formerly known as “bovine aortic arch” (with an incidence of up to 27%)) or one of multiple pathological conditions. The present study aimed to establish the feasibility and impact of prenatal anatomic arch variants’ diagnosis. A retrospective study of 271 fetal second- and third-trimester anomaly scans was performed in our tertiary center. Examinations that evaluated the sagittal aortic arch were included and the branching pattern was assessed. Additionally, a literature data search based on the terms “common origin of innominate artery and left common carotid artery”, “bovine arch”, “bovine aortic” and “aortic arch anomalies” was performed. Results that referred to prenatal AAAV were retained and the papers evaluated. In our study, the AAA incidence was 1.93%, with 4 out of 5 cases being arch type B. All cases had minor associated conditions but a good postnatal outcome. An anatomic aortic variant with a common IA and LCCa prenatal diagnosis was found in a small number of studies; most of the cases described in pediatric and adult series were related to cardiac surgery for stenting, aneurysm or thoracic-associated diseases. The incidence of AAAV varied from 6 to 27% depending on the population studied (highest incidence in African individuals). The variant was highly associated with aortic dissection, pulmonary and cerebral embolism and increased risks of incidents during surgery. Diagnosing AAAV during a routine anatomic scan is feasible and diagnoses can be made when anomaly scans are performed. Awareness of the condition is important for postnatal surgery when other cardiac anomalies are found; this can prevent accidents with simple changes to the patient’s lifestyle, and, in the case of surgery, means we can adopt the correct surgical approach.

## 1. Introduction

Fetal aortic arch development starts early in pregnancy (around day 41 of gestation) from the primitive III-d and IV aortic arches and reaches its final circulation pattern at about 57 gestation days, with the process being guided by the neural crest cells [1].

Many variants of the final aortic arch have been described, some without pathological meaning, i.e., anatomic variants (bovine aortic arch), and others that can affect the fetal and neonatal outcome (double aortic arch, interrupted aortic arch, aortic coarctation) [2].

Laterality anomalies includes the right aortic arch (RAA) with left arterial ducts or in association with the right arterial duct. As for other cardiac anomalies, the awareness that the anomaly can be easily detected at the level of 3 vessels view when the arch is coursing to the right of the trachea, increased dramatically the number of detected RAA [3]. Recent systematic review and meta-analysis prove that RAA is associated with an increased risk for congenital heart disease in about 30% of cases, extracardiac defects in 14.1% of cases and moreover to chromosomal aberration as 22q11del (di George syndrome) (ranging from 1% to 6%) regardless of the laterality of the ductal arch [4,5].

Another branching anomaly which accounts for 0.4% of the population consists of an abnormal right subclavian artery (arteria lusoria). In this situation four arteries are emerging from the left aortic: the right common carotid, the left common carotid, the left subclavian, and the aberrant right subclavian artery—ARSA. The late is arising most distally from the aortic arch, and its course is found behind the esophagus and the trachea, to the right upper arm. ARSA was first described in fetuses by Chaoui and isolated ARSA has generally a favorable prognosis. However relative risks of associated chromosomal and cardiac defects increase when ARSA is present (still with rather low absolute risks). Additional testing is indicated based upon a comprehensive case by case assessment [6,7].

In variants that need prompt postnatal intervention, adequate prenatal detection is of paramount importance for allowing for the proper birth setting to be initiated in a tertiary center with multidisciplinary teams’ collaboration.

**Figure 1:**
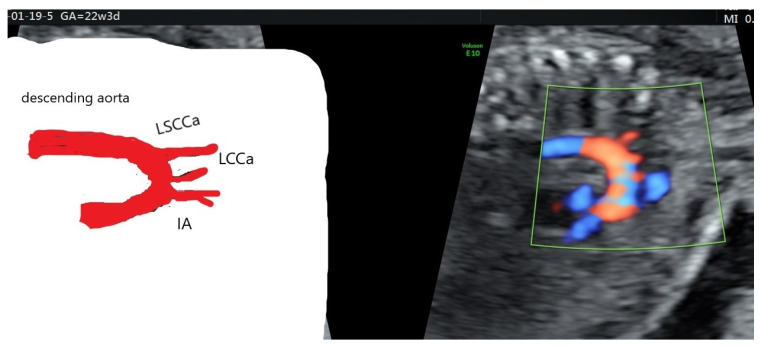
Schematic figure of normal (type I) aortic branching. Sagittal section of fetal thorax, spine anterior. Dao—descending aorta, IA—innominate artery (brachiocephalic trunk), RSCa—right subclavian artery, RCCa—right common carotid artery, LSa—left subclavian artery.

Common aortic branching, accounting for 84% of the population, consists of three main vessels that arise in the following order from the origin to the descending aorta: innominate artery (or brachiocephalic trunk), from which common right carotid (RCCa) and right subclavian artery (RSCa) branch further; common left carotid artery (LCCa); and left subclavian artery (LSCa) [2,8] (Figure 1).

**Figure 2:**
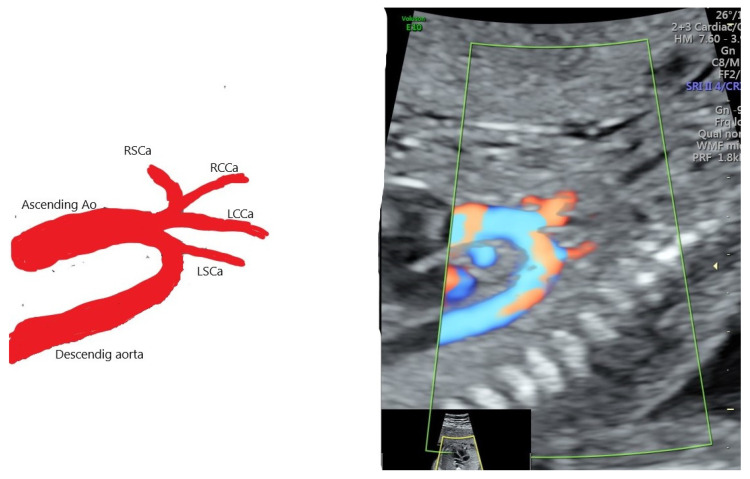
Schematic figure of AAAV type A IA—innominate artery as a common trunk from which three vessels is branching LCCa, RSCa and RCCa.

**Figure 3:**
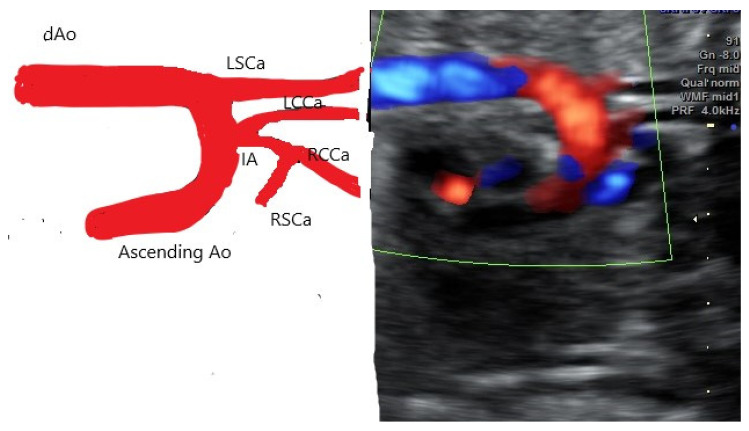
Figure 2 Schematic figure of AAAV type B IA—and LCCa are arising in the same spot the same spot. IA divides furtherly into RSCa and RCCa. This is most common AAAV variant.

An anatomic aortic arch variant (AAAV) is a different type of aortic branching, considered as the second most frequently encountered, but the pathological significance in prenatal life is not clear. In the AAAV pattern, only two vessels arise from the aortic arch: a common trunk for RCCa, RSCa and LCCa, and, at a variable distance from it, LSCa [9]. There are two subtypes of AAAV: A, where the LCCa is a branch from the common trunk, and B, where both IA and LCCa originate in the same spot (Figure 2 and Figure 3).

The AAAV incidence ranges from 6 to 27% in postnatal, mainly adult examination series [2,8,9,10,11,12,13,14,15]. The greatest incidence was reported in a recent meta-analysis of the adult African population [14].

Prenatal series of the AAAV pattern are scarce, and imaging of the longitudinal view of the aortic arch is not included in scan protocols [14,15].

The present study aimed to review the significance of the anatomic aortic arch variant with a common origin of the brachiocephalic trunk and left common carotid artery in prenatal examinations. We excluded laterality anomalies (RAA with left DA or right DA), double aortic arch, interrupted aortic arch, aortic coarctation. The study was prompted from a series of cases in our practice, to underline the importance of prenatal identification when we consider the potential association with other cardiac or extra-cardiac conditions.

## 2. Material and Methods

We retrospectively reviewed ultrasound examinations performed in our center over a 12-month period. We scanned 271 pregnancies in the second and third trimesters according to the national protocol for anomaly ultrasound evaluation, between 20–24 weeks and 30–32 weeks of gestation. Although the protocol does not include, for screening reasons, a sagittal view of the aortic arch, as a tertiary educational center, we obtain that image systematically in the second trimester and whenever possible in the third trimester. All examinations were performed by two certified materno-fetal specialists on a Volusson E8-10 machine. The standard acquired images were obtained with a volumetric RAB 4–8 MHz abdominal transducer and include: the four-chamber view, left and right outflow tracts, three vessels and trachea view. Additionally, we obtained sagittal views showing the aortic arch, ductal arch and bicaval picture. The sagittal view was obtained at the fetal thoracic level with the spine anterior or posterior, initially in B mode, and then the Doppler color or HD flow mode was applied. In order to display the sagittal section of the thorax and the aortic arch the following steps should be followed: first asses the four-chamber view in transversal thoracic section, then rotate the probe clockwise and tilt toward the left fetal shoulder in order to obtain the long axis view (ventriculo-arterial connections and crossing of the Ao and MPA can be assessed), slightly rotation of the probe clockwise will display the aortic arch view. The curvature of the aortic arch is more pronounced than the ductal arch curvature and also has the typical emergence of the head and neck vessels. The first arising vessel (closest to the Ao emergence) is the innominate artery (brachiocephalic truck) that branches into the right subclavian artery and right common carotid artery. The second branch of the aortic arch is the left common carotid artery and the most distal vessel arising is the left subclavian artery. The aortic arch is usually to the left of the trachea and in its concavity the left pulmonary artery can be seen. We excluded the images that were unclear or when arch branching could not be clearly assessed. The images and clips are stored for internal and external auditing purposes and to be used as evidence, if necessary. 

Ethical approval from the unit’s Ethical Committee was obtained for the study.

In addition to this, we performed a narrative review on database search with the terms: “common origin of innominate artery and left common carotid artery”, “bovine arch”, “bovine aortic” and “aortic arch anomalies” in PubMed, Embase, Scopus, ScienceDirect, Web of Science and Cochrane. Since “bovine aortic arch” is often used in the literature as a synonym for the aortic arch variant with a common origin of the innominate artery and left common carotid artery, but is considered a misnomer since the arrangement of the branching in cattle is different [7], we included this term in our search. No language restrictions were applied and all types of items (cases reports, systematic reviews, literature reviews, etc.) were accepted. After excluding duplications, the returned items were examined and included in the study only if they referred to the prenatal diagnosis of AAAV. 

## 3. Results

From the 271 examinations performed, 12 were excluded because of a lack of clarity or absence of the aortic arch view in the sagittal plane. Five fetuses were found to have an aortic arch variant with an incidence of 1.93%. The main characteristics of the pregnancies with fetal AAAV are depicted in Table 1.

The mean maternal age was 34 years and mean gestation age at diagnosis was 26 + 2.4 weeks.

**Figure 4:**
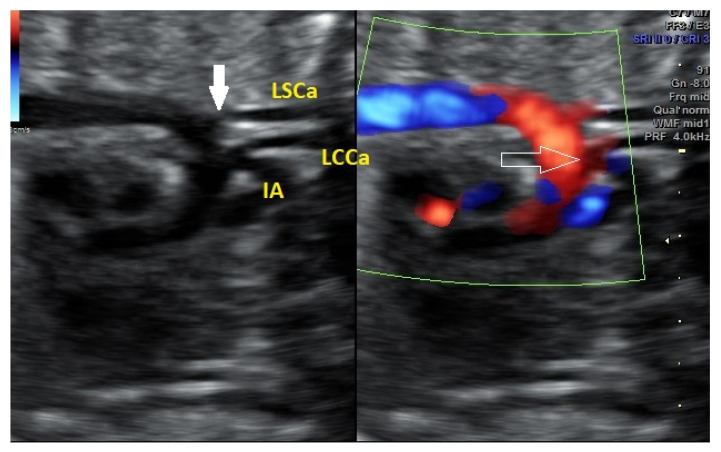
AAAV type B at 21 weeks, B mode and Doppler colour side by side. Note the common origin of IA and LCCa.

**Figure 5:**
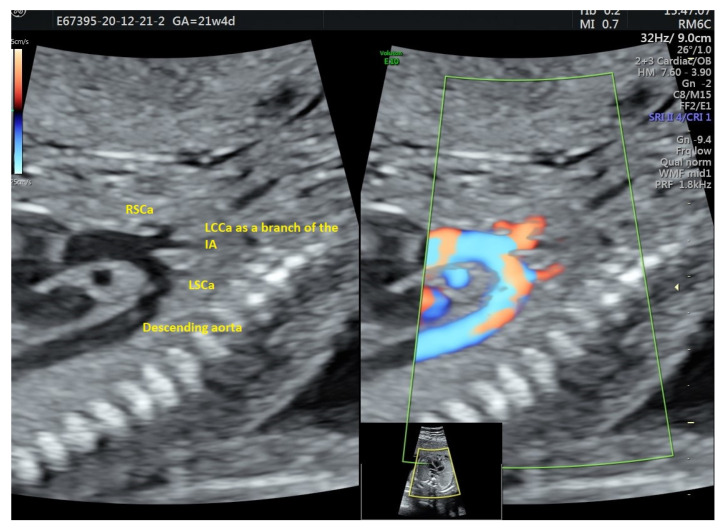
AAAV type A at 21 + 4 weeks, b mode and Doppler color, the common origin of IA and LCCa is more obvious on Doppler mode.

**Figure 6:**
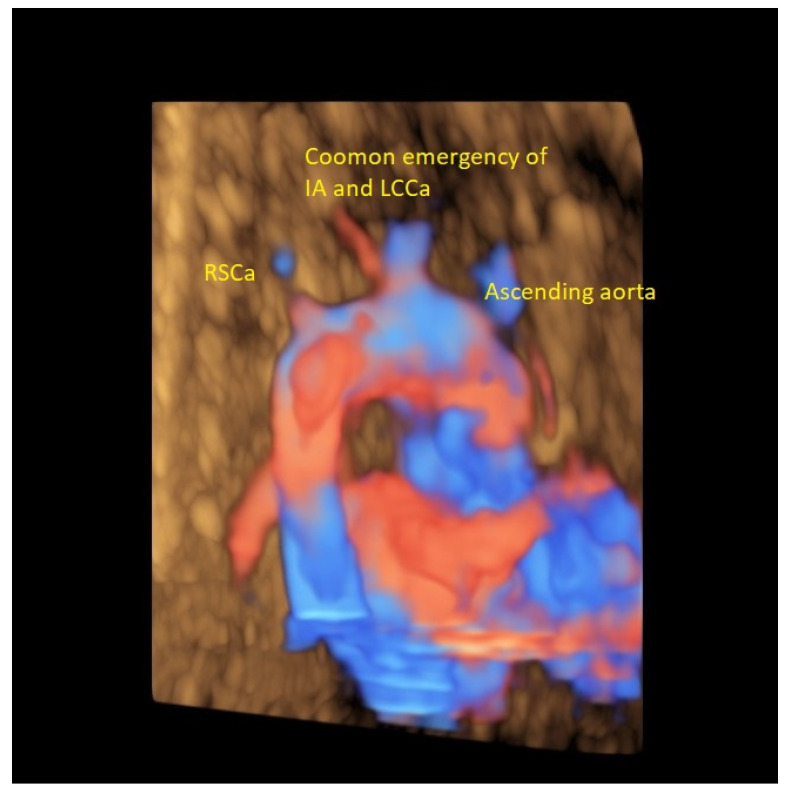
3D color image rendering of a AAAV type B.

Only one case of AAAV was type A (distal branching of the LCCa); the rest were type B (Figure 4, Figure 5 and Figure 6).

Maternal conditions were difficult to correlate with the incidence of AAAV but we noticed that, in one case, echocardiography was mandatory considering the previously affected child (Noonan sdr), and despite a minor association, the CMA proved normal.

The immediate postnatal outcome of the babies was uneventful in all five cases, and the pediatric cardiologist confirmed the AAAV variant in all cases. In one case. we overlooked a small outlet VSD that had no impact on the cardiac hemodynamics.

**Figure 7:**
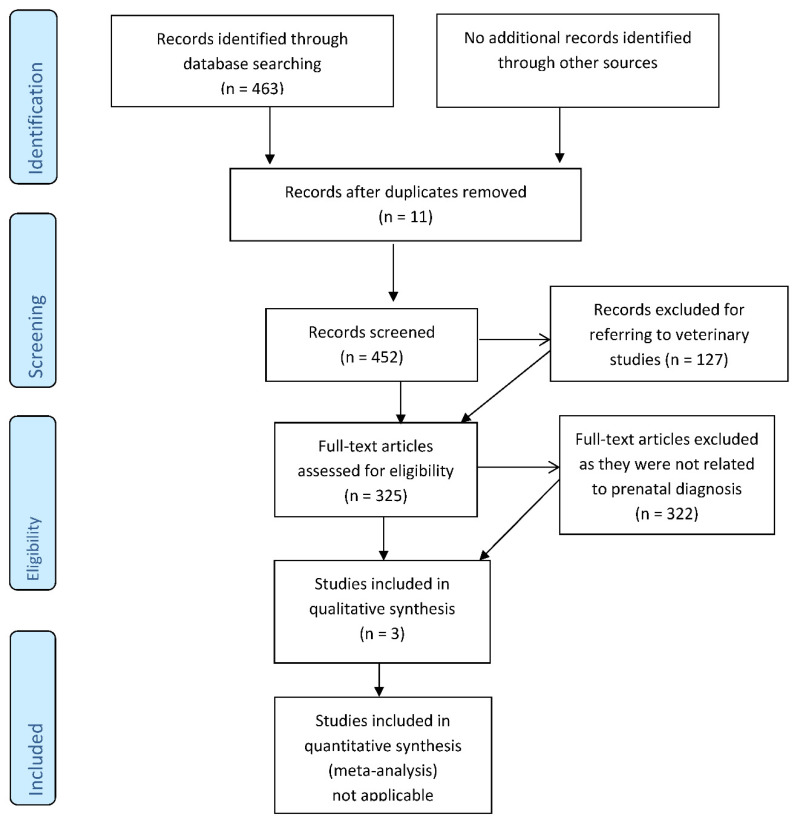
Flow diagram of the research.

The database search provided 463 results for the searched terms. The vast majority provided data from surgery or radiologic intervention in adults for stenting or valve repair and problems encountered whenever anatomic variants were present, associated with aortic dissection and associated pulmonary and cerebral embolism. Only three reported prenatal cases or studies on prenatal series [14,16,17] and one abstract [18] (Table 2 and Figure 7) all the 3 papers were written in English; 127 items referred to veterinary studies.

Five systematic reviews and meta-analyses were found, all addressing the incidence of branching or surgical implications of the anatomic aortic arch variant [3,9,19,20,21] but none addressed the prenatal diagnosis.

## 4. Discussion

The aortic arch is one of the most important vessels in fetal and postnatal life, and it is prone to a wide spectrum of anomalies with or without pathological impacts. Considering the complexity of the embryological evolution—the presence of six pairs of aortic arches (but only arches three, four and six contribute) that will suffer apoptosis, further branching and spatial evolution, the early onset in the embryonic period when those processes occur and the multitude of local, environmental and genetic factors involved—aortic arch anomalies can be a trigger for the presence of other structural or chromosomal or genetic anomalies [22,23].

The appearance of a definitively normal (type I) left-side aortic arch depends on complex evolving structures including: cells of the ectomesenchimal neural crest, ectoderm, mesoderm and exocellular matrix. Many genes have been implicated in the process, and HOXA genes (HOX 1, 2, 3) have been demonstrated in animal studies to be involved in remodeling the pharyngeal arch arteries. Malfunctioning of those genes expressed throughout cardiac development can explain complex deformative conditions of the heart [24].

Recent studies suggest that multiple factors are involved in remodeling the aortic arch: growth factors, metalloproteinase and receptor signaling pathways. Among those, ADAMTS, a group of 19 secreted metalloproteinases, are involved in growth, branching, differentiation and valvular formation and play a decisive role in the development of the aortic arch and common carotid arteries [25]. Those factors are responsible not only for cardiovascular development but also for diseases such as atherosclerosis, pulmonary arterial hypertension and aneurysm. That can explain the association between AAAV and aortic dissection and aneurysm, as seen in adults [26,27,28].

Knowledge of the anatomical aortic arch variant can be important whenever association with other anomalies are found, and surgery can avoid accidental lesions of the aortic branches [29,30].

AAAV can be associated with genetic syndromes, according to Turner [31]. Considering the increased association with aneurysm dissection and surgical incidents in common-origin IA and LCCa, there is no need for surgical correction in cases of AAAV, but physicians should be aware of the risks implied by the presence of the variant, especially when arch or cardiac anomalies are found (frequent in Turner sdr) [32].

Associations with aortic coarctation when surgery is needed in the neonatal or infant period are important to determine, as well. Some authors have postulated that the surgical results could be suboptimal or compromised in AAAV-associated coarctation due to technical limitations (reduced distance for vascular clamp placement). In such cases, the prenatal diagnosis of the coexisting condition could save time and allow a proper management plan to be formulated before birth [27,33].

Prenatal diagnosis of AAAV can be easily made by obtaining a sagittal section of the fetal thorax with both the ascending and descending aorta and so the branching can be observed [14]. In our study, we could obtain accurate aortic arch images from 82% of the routine scans, following the algorithm described in Material and Methods. The whole process can add to the examination time since there are factors that can reduce visibility such as anterior position of the fetal spine and shadowing from the ribs and vertebral body, the presence of fetal arms in front of the thorax when the spine is posterior or active fetal moving. It is time-consuming, but once the image is captured properly, the branches are easy to establish. In their study, Goldsher recorded better aortic arch visualization (92%), possibly due to better training or more time allocated to the examination. Despite the above-mentioned difficulties in acquiring a proper image, we think that the effort is worthwhile in all cases as it can demonstrate besides the aortic arch variant also arch anomalies such as aortic coarctation, interrupted aortic arch, right aortic arch, etc.

Compared to our study, they also demonstrated a higher incidence of AAAV (4.8%), which could be explained by our small cohort of patients and also by the high homogeneity of our population, which was almost exclusively Caucasian (proven to have the lowest frequency of AAAV) [10,14,18].

The literature mainly provides data from adult studies—which could present a bias versus the real incidence of AAAV—where patients were identified by the presence of comorbidities and diagnosed using computer tomography scans and other radiological investigations, or even in post-mortem series [1,10,34,35,36,37].

Many studies associate AAAV with aortic dilatation, aneurysm, stroke, cerebral and pulmonary embolism and valvular disease. Cardiac and vascular surgeons are the main healthcare professionals concerned with AAAV since it can make surgery more challenging and prone to incidents such as blunt traumas or even aortic tears. The approach to grafting or stenting with AAAV is often more difficult, needing a brachial or radial approach instead of femoral [19,21,30]. Moreover, AAAV itself can be considered a marker for aortic and thoracic disease, produced by an increased velocity of flow through the aortic arch, with fewer branches to derivate the flow, The increased size of common-origin IA and LCC can contribute to a sheer force that induces aneurysm and can lead to aortic dissection [24].

The association of pathological conditions, in our study, could not be properly evaluated, though 80% of the fetuses had associated conditions not directly related to the AAAV. As Goldsher also found, a minor fraction of the fetuses with AAAV had serious associations (4–5%) [15].

In the literature, there was only one report of a severe association of AAAV with double left-inlet ventricle and pulmonary stenosis in the prenatal period [17] and one of a newborn with multiple heart anomalies (dextrocardia, tetralogy of Fallot and BA) that raised surgical challenges for the repair [27]. In both cases, the gravity of the outcome was not dictated by the presence of AAAV, but surgery was more difficult because of its association.

The hemodynamics of the AAAV could differ from that of a type-I aortic, arch as postulated in the paragraph above, according to adult studies [25]. We did not evaluate the velocities in the different segments of the fetal circulation as Clerici did in his study. He compared the epiaortic hemodynamic parameters in 472 fetuses, of which 45 had AAAV. The vascular sites assessed to compare the flow were the IA, LCC artery, LSC artery and MCA for both groups of patients. The study found a significantly higher peak systolic velocimetry in all areas with normal branching compared to the AAAV variants. The hemodynamic in MCA was comparable in both groups with no significant difference, meaning that the cerebral territory was unaffected by the different branching types [17]. The results are not transferable to the hemodynamics in adult life since the arrangement of ventricular functioning changes: in fetal life, the two ventricles work in parallel and the main cardiac output is the right ventricular one, transferred to the systemic circulation via the arterial duct. It would be interesting to extend the experience and confirm these findings in a larger cohort of fetuses.

The limitations of our study are the small sample of pregnancies investigated and the retrospective nature of the evaluation. To obtain significant data concerning the real incidence of AAAV in our prenatal population, a larger cohort of pregnancies must be assessed. We had the advantage of studying a homogenous population, but interrogating a larger sample of pregnancies could produce findings that are more representative of the real AAAV incidence in the population. Moreover, introducing the sagittal view of the aortic arch into routine second-trimester scans could be beneficial for prompting a more detailed evaluation of the fetal heart in cases where the scan identifies an anomaly. Further studies including larger population groups will establish the real incidence of AAAV in our population and will provide evidence for extended systematic cardiac evaluation.

## 5. Conclusions

An anatomic aortic arch with a common origin of IA and LCCa is a variant of the aortic arch branching with an incidence that can range in adults from 4.8% in Caucasians to 27% in the African population, but the prenatal incidence is not yet clear. Diagnosing AAAV during a routine anatomic scan is feasible and can be carried out by including the sagittal section of the thorax with the typical image of the branching aortic arch in anomaly scans. Although studies do not indicate an increased frequency of other cardiac anomalies, whenever other malformations or syndromes are detected (i.e., coarctation, right aortic arch, Turner syndrome, del 22q11, etc.) that require postnatal surgery, knowledge of the aortic arch type is beneficial for avoiding incidents and difficulties in surgery. Moreover, as AAAV is considered a marker for many thoracic and aortic diseases in young people and adults, awareness of the condition can prevent accidents by making changes to the patient’s lifestyle or can help in the case of surgery, as the surgeons can adopt the correct approach.

## Figures and Tables

**Table 1:** The main characteristics of the pregnancies with fetal AAAV.

Case Maternal Age	Gestational Age at Diagnosis	Parity	Type of AAAV	Significant Maternal Conditions	Associations	Genetic Testing	Outcome at Birth
31	21 + 4	1	type B	none	none	ND	Normal neonatal cardiac scan confirmed AAAV
43	23 + 2	1	type B	IVF pregnancy	FGR	NIPT (low risk)	Normal small-outlet VSD postnatal scan confirmed AAAV
28	30 + 2	3	type B	Previous fetus with Noonan sdr	Megacysterna magna, weird profile with long philtrum	Amnio 46XY	Normal neonatal cardiac scan confirmed AAAV
32	24 + 1	2	type B	none	Bilateral hydronephrosis	ND	Normal neonatal cardiac scan confirmed AAAV
36	32 + 3	1	type A	MBI 36.5	Polyhydramnios	NIPT (low risk)	Premature birth neonatal cardiac scan confirmed AAAV

**Table 2:** Results of literature.

Study, Author Type Year	Number of Reported Patients	Gestational Age at Diagnosis (Wks.)	Associated Anomalies	Fetal Gender	Genetic Assessment	Follow-Up Image Findings	Pregnancy Outcome	Neonatal/Post Termination Diagnosis
Goldsher YW 2019 Retrospective	20 4.8%	15–40 weks	No	NR	NP	NR	Normal	NR
Pinto A2018 Retrosepective	13 2%	Average 23 wks	7 53.8% VSD, HB, ARSA, DIRV, D-TGA Polidactily	NR	9 normal 4 NP	NR	Nr	4 neonate with major cardiac anomalies requiered surgery
Clerici G 2018 Prospective descriptive	45 6.06%	21–39 wks	NR	66.7% males	Not performed	Velocimetry Differences from Type I AA	Normal	Non reported
Alghamadi MH 2009	Case report	No specifid	DILV Sigle coronary artery Bovine aortic arch B	Male	Normal	NR	Normal	Anomalies confirmed, surgery after DA patency mentained by prostaglandin infusion Scheduled for Fontan palliation

## Data Availability

The data presented in this study are available on request from the corresponding author.

## Abbreviations

AAAV	anatomic aortic arch variant
IA	innominate artery
BCT	brachiocephalic trunk
RCCa	common right carotid
RSCa	right subclavian artery
LCCa	common left carotid artery
LSCa	left subclavian artery
